# A taxonomy for community-based care programs focused on HIV/AIDS prevention, treatment, and care in resource-poor settings

**DOI:** 10.3402/gha.v6i0.20548

**Published:** 2013-04-16

**Authors:** Beth Rachlis, Sumeet Sodhi, Barry Burciul, James Orbinski, Amy H.Y. Cheng, Donald Cole

**Affiliations:** 1 Global Health Division Dalla Lana School of Public Health, University of Toronto, Toronto, Canada; 2 Department of Family and Community Medicine, University of Toronto, Toronto, Canada; 3 Dignitas International, Toronto, Canada; 4 University Health Network, Toronto, Canada; 5 Centre for International Governance Innovation, Waterloo, Canada; 6 St. Michael's Hospital, Toronto, Ontario; 7 Division of Emergency Medicine, Department of Medicine, University of Toronto, Canada

**Keywords:** community-based care, HIV, resource-limited settings, review, taxonomy

## Abstract

Community-based care (CBC) can increase access to key services for people affected by HIV/AIDS through the mobilization of community interests and resources and their integration with formal health structures. Yet, the lack of a systematic framework for analysis of CBC focused on HIV/AIDS impedes our ability to understand and study CBC programs. We sought to develop taxonomy of CBC programs focused on HIV/AIDS in resource-limited settings in an effort to understand their key characteristics, uncover any gaps in programming, and highlight the potential roles they play. Our review aimed to systematically identify key CBC programs focused on HIV/AIDS in resource-limited settings. We used both bibliographic database searches (Medline, CINAHL, and EMBASE) for peer-reviewed literature and internet-based searches for gray literature. Our search terms were ‘HIV’ or ‘AIDS’ and ‘community-based care’ or ‘CBC’. Two co-authors developed a descriptive taxonomy through an iterative, inductive process using the retrieved program information. We identified 21 CBC programs useful for developing taxonomy. Extensive variation was observed within each of the nine categories identified: region, vision, characteristics of target populations, program scope, program operations, funding models, human resources, sustainability, and monitoring and evaluation strategies. While additional research may still be needed to identify the conditions that lead to overall program success, our findings can help to inform our understanding of the various aspects of CBC programs and inform potential logic models for CBC programming in the context of HIV/AIDS in resource-limited settings. Importantly, the findings of the present study can be used to develop sustainable HIV/AIDS-service delivery programs in regions with health resource shortages.

The Joint United Nations Program on HIV/AIDS (UNAIDS) estimated that globally approximately 34.2 million people were living with HIV in 2011 (1). Sub-Saharan Africa remains the most heavily affected region (2, 3). While coverage remains low in many settings (4, 5), access to highly active antiretroviral therapy (HAART) has increased in recent years, with more than 6 million people in low- and middle-income countries receiving treatment in 2010 (1). Increased availability of antiretroviral therapy (ART) has not only dramatically increased survival but has also changed the clinical management of HIV (6) requiring increasing numbers of trained health workers to effectively deliver treatment (7).

The chronic shortage of health workers amplifies this challenge. According to the Global Health Workforce Alliance, 1.5 million more health care workers are needed in sub-Saharan Africa alone just to be able to provide basic health services (8). Furthermore, high healthcare costs and lack of adequate healthcare infrastructures further decreases the ability to deliver HIV services in resource-limited regions (9–12). Decentralizing HIV services through community-based approaches has been used in many regions with limited healthcare resources to overcome the challenges imposed by the scale of the HIV/AIDS epidemic (13–15).

There is currently no single consensus definition of community-based care (CBC) (16). It can mean different things, have different uses, and play different roles for different actors (e.g. program implementers, policy makers, donors). Within the context of HIV/AIDS, CBC includes ‘all AIDS activities that are based outside conventional health services (hospital, clinic, and health centre), but which may have linkages with the formal health and welfare sector, and which address an aspect of the continuum of care from the time of infection through to death’ (17). The World Health Organization (WHO) defines CBC as care that ‘the consumer can access nearest to home, and which encourages participation by people, responds to the needs of people, encourages traditional community life, and creates responsibilities’ (18). Home-based care (HBC) and CBC are two closely related concepts. HBC can be defined as ‘the provision of services by formal and informal caregivers in the home’ (18). As such, HBC is often an integral part of CBC. While there are common components of CBC programs across settings (19, 20), the specifics of a given model depend on contextual factors, including national policy, non-governmental organizations’ (NGO) missions, and available resources.

The use of community health workers (CHWs) was integrated into primary healthcare reform in the 1970s (21, 22). As the HIV pandemic took hold in sub-Saharan Africa, CHWs were frequently responsible for disease monitoring and patient support tasks (18, 23). In the 1990s, CBC programs that addressed various aspects of the HIV/AIDS continuum of care (e.g. testing and counseling services, medical and social support) were introduced in countries affected by the epidemic (15, 23).

With the scale-up of ART in the global South came new challenges: delivering treatment to those in need and providing lifelong care for those now on life-extending treatment (24–27). To maximize access to ART with scarce health professional resources, health planners have shown renewed interest in the potential role of community-based programs and CHWs in ART monitoring. Physician-centered models in many settings have given way to those reliant on trained non-physician health workers (13, 26, 28). Nurse-driven ART in Lesotho (28), the use of community-based Health Surveillance Assistants (HSAs) to dispense ART and monitor stable patients in one region of Malawi (29), and community care coordinators in Kenya (13) are examples of such ‘task-shifting’. Individual CBC programs have been shown to increase access to essential services for people living with HIV/AIDS (PLWHA) and their families (30, 31) through the mobilization of community interests and resources as well as the development of appropriate tools, including training and referral, to integrate these with formal health structures (15, 19). Available data suggest that CBC programs can promote awareness while reducing stigma, reduce the burden on the primary care system, and ultimately decrease the incidence of hospitalization (32, 33). In the context of treatment scale-up, some CBC programs shifted their focus in order to encourage optimal adherence to ART, supporting better treatment outcomes. Evidence suggests that at least in some settings, viral load suppression and adherence may be improved in community-based programs relative to clinic-based care (7, 34).

With ART coverage increasing in many settings, understanding the role of CBC programs becomes more and more important (16). Related to this is the need to understand how CBC programs can target and support vulnerable and marginalized populations that continue to face barriers in terms of knowledge about HIV and its prevention, treatment, support, and care, and in terms of access to these services (35, 36). Rollout of ART to vulnerable populations may require alternative approaches that work best when communities and community resources are engaged in the process (7). Significantly, there is evidence suggesting that CHW programs have not always been successful (30). The mix of activities in CBC programs make them difficult to study, limiting the explicitness and comparability of what have, to date, been primarily program-specific evaluations (16). While such evaluations are important for providing evidence of programs’ effectiveness, they need to be accompanied by analyses that identify the conditions necessary for effective and sustainable program implementation (15, 37). A 2012 review of community-based organizations (CBOs) that sought to identify and conceptually map existing peer-reviewed literature related to the characteristics of CBOs in the health sector noted significant gaps in our understanding of CBOs in low- and middle-income countries (16). Furthermore, an absence of explicit conceptual frameworks in such settings continues to limit our ability to understand and study health policy (38). We sought to develop a taxonomy of CBC programs focused on HIV/AIDS in resource-limited settings in an effort to identify and describe their key characteristics, uncover any gaps in programming, and highlight the potential roles they play.

## Present investigation

### Methods

We conducted electronic searches of health bibliographic databases including PubMed via Medline (from inception to December 2011), CINAHL (from inception to December 2011), and EMBASE (from inception to December 2011). Our search terms were ‘HIV’ or ‘AIDS’ and ‘community-based care’ or ‘CBC’. Additional articles were sought by examining the bibliographies of relevant articles. For the gray literature, we used the same search terms in Google and Google Scholar to identify program descriptions, study reports, and publications of official materials issued by non-governmental organizations, government health services, and international agencies. Site-specific searches of the UNAIDS and WHO websites were also undertaken.

In the present study, only CBC programs operating in resource-limited country settings were included. We defined resource-limited as a low-income or middle-income country using the World Bank Country Classification (39). As we believe this to be the first comprehensive review focusing on CBC programs related to HIV in resource-limited settings, we used a broad definition of CBC. It encompassed any self-identified CBC program focused on HIV prevention, treatment, care, and support that reported any level of community participation in the planning and/or delivery of care. However, in order to develop the taxonomy, our inclusion criteria required that programs provided adequate descriptions on program origins/mission, structure, populations served, and day-to-day activities.

For analysis, we undertook an inductive and iterative process to develop our taxonomy of CBC programs. First, Beth Rachlis (BR) conducted the search of potentially relevant CBC programs (as case studies) and determined which programs provided data based on our inclusion criteria. Next, BR extracted all descriptions from each included CBC program. From the gathered information and using an inductive approach, BR and Sumeet Sodhi (SS) sorted the data and descriptions extracted from each individual program into broader categories, in an iterative process. Finally, we sought to organize the information into distinct and mutually exclusive categories (e.g. program scope vs. program operations). For content validation, we ensured that the information described and extracted from most individual programs could be categorized as per our developed taxonomy.

## Results

Our initial search of the literature returned 2,524 citations, of which 83 were considered potentially relevant. Of these, 64 were excluded because they did not describe a CBC program (*n*=45), were not based in a resource-limited setting (*n*=10), or were duplicates (*n*=9). We updated our literature search in February 2012 and found two additional programs that were relevant. Therefore, 21 CBC programs (14, 19, 30, 34, 40–53) met our inclusion criteria and were included. Table 1 describes our taxonomy and Table 2 describes the nature of the included CBC programs.

**Table 1:** Taxonomy of community-based care (CBC) programs

Category	Item	Working definitions
1. Region		Geographic location (e.g. region, country)
2. Vision	Vision, mission, values	Overall mandate, goals or other broad rationale
3. Characteristics of target population	Level of urbanization	Intra-country locations of client-patient populations (e.g. urban, peri-urban, rural)
	Targeted population	Primary population of interest (e.g. PLWHA)
	Vulnerable groups included	Inclusion of other affected populations (e.g. migrants, men who have sex with men)
4. Program scope (services provided)	Location of services	
	At home	Service provision and care in client-patient's home
	In the community, outside of the home	Care outside of the home in community agencies or sites
	Both in and outside of the home	Care provision in both settings
	Types of Services	
	HIV prevention activities	Services, including condom distribution, prevention education
	HTC	Services of HIV testing and counseling
	PMTCT	Provision of antiretrovirals for the prevention of mother-to-child transmission of HIV
	ART	Provision of antiretroviral therapy and follow-up care (e.g. adherence monitoring and support)
	Medical and nursing support	Basic medical care, including treatment and management of STI (sexually transmitted infections) and OI (opportunistic infections)
	Nutrition support	Nutritional support, including provision of food supplements
	Palliative care	Care and support for terminally ill patients
	Financial support	Income generating activities or provision of funds (e.g. community funds).
	Material support	Technical support (e.g. training, lifeskills) or provision of material supplies (e.g. soap).
	Psychosocial support	Social support and counseling
5. Program operations	Extent of community engagement (in operations)	Community involvement in overall operations (e.g. decision making, planning, accountability)
	Reach	Overall reach or coverage geographically (e.g., how many sites the program has) or by population (e.g. how many patients of what kind are served)
	Service delivery model	Primary mechanism used to achieve coverage (e.g. decentralization)
	Embeddedness in formal health and social structures	Degree of entrenchment within existing structures (e.g. number and types of partnerships or collaborations)
6. Funding models	Sources of funds	Revenue – who pays what (i.e. donations, funders), diversity
	Fund utilization	Use of funds (e.g. what money is needed and used for what purposes)
	Budget and financial mechanisms	Spending plan (e.g. how budgets are set, how spending is regulated and monitored)
7. Human resources	Organizational structure	Hierarchical or horizontal structure and responsibilities of individuals and groups (e.g. advisory boards)
	Staff composition (volunteers, paid staff)	Staff make-up and positions of individuals within team
	Training and mentorship	Incorporation of training activities and opportunities for mentorship
	Management and supervision	Extent to which staff are managed and/or supervised
8. Sustainability	Availability of ongoing funding	Promises or plans for ongoing financial support
	Staff retention strategies	Ability to retain staff though the use of incentives (e.g. compensation)
	Replicability of program	Adaptability and ability to be reproduced in a new setting
9. Monitoring and evaluation	Evaluation strategies	Mechanisms to determine overall success and impact (i.e. how programs are being evaluated)

**Table 2:** Program descriptions by taxonomy categories

CBC program	Region	Vision	Characteristics of target populations	Program scope	Program operations	Funding models	Human resources	Sustainability	Monitoring and evaluation
AIDS Foundation Est. 1988 (first registered NGO in South Africa) (Source: AIDS Foundation South Africa website)	Africa: South Africa	To be the leading organization supporting CB developmental interventions in the HIV/AIDS epidemic	– Members of selected CBO targeting HIV prevention, Culture and Health, HBC, Care for OVC, Poverty alleviation. – target members of sexual minority populations including individuals who identify as Lesbian Gay, Bisexual, Transgendered	– Community – Training for HBC, provides program management, report writing, research and program monitoring & evaluation	– Links and places donor funds with selected CBO – Mentoring and capacity building – Partners include the AIDS Consortium, Treatment Action Campaign, Basic Income Grant Coalition, KZN Civil Society Network, HIVAN – Includes 94 partner organizations local and at national level – 7 provinces in South Africa – Supporting 83 CBO	Donors include: – Bernard van Leer Foundation, Ford Foundation, Elton John AIDS Foundation, Artist for a New South Africa – Norwegian Church AIDS, and Bread of the World – Canadian and Swedish International Development Agencies, Irish AIDS	– Staff of 31 – Governed by Board of Directors (6 board members)	– Board of Directors maintain organization focus and accountability – reputation for professionalism	– Monitor and evaluate, identify assistance needs, quality assurance, set standards – Examine outcomes at end of three-year cycles – Conduct research and evaluation – Annual reports
Catholic Diocese in Ndola Est. 1993 (Source: Fikansa, 2005) New Source: Stop AIDS Now 2009	Africa: Zambia	To operate on a non discrimination basis	– PLWHA, families, OVC, Community Care Givers – Urban and rural districts	– Home, community – HTC, ART, HBC, medical care, nursing care, TB, psychosocial support, pastoral support, welfare support for households, IGA, PMTCT, microfinance	– Provision of care incorporates community volunteers and integrated through local hospitals, partnerships with community resources to build capacity – 13 home-based care centers – 44 shanty compounds – 5 towns – interdominational approach		– Coordinator, and nurses oversee community volunteers – Training and follow-up courses	– Linked to National AIDS Council Strategic Framework	– Program monitoring, reporting (on a half-yearly basis
Centre for Positive Care Est. 1993 (Source: Centre for Positive Care website)	Africa: South Africa	To reduce the spread of STI, HIV/AIDS and improve the quality of life	– PLWHA, OVC, CBO staff	– Home, community – HIV prevention & risk reduction programs, VCT, PTMCT, HBC, STI treatment, palliative care, lay counseling, spiritual support, peer education, train family, material supplies (e.g. seeds for gardens)	– Incorporates community leaders, community members trained as peer-educators – Partners with local, national, international organizations – 4 districts – Community driven approach	Donors include: – Department of Health (South Africa,), European Union – Bristol-Myers Squibb Foundation – Nelson Mandela Children's Fund, Family Health International, National Development Agency, Christian AID, Reggio nel Mondo, AusAID, OXFAM, Save the Children UK, VSO	– Board of Directors-staff and local community members, including local magistrate – Volunteers supervised by coordinators – Mentorship to lay counselors	– Stipends and incentives for retention – Continued training through a learning institution – Training of local volunteers to carry on activities	– An evaluation was performed by Nelson Mandela Children's Fund – Evaluation on pediatric palliative care
Chirumhanzu HBC project Est. 1994 (Source: Family Health International)	Africa: Zimbabwe	To meet the needs of PLWHA and their families and to provide necessary info, skills, care, & support	– PLWHA, family members, caregivers – Rural area	– Home, community – Prevention campaigns, HBC, support groups, life skills, drama clubs, recreational activities, awareness campaigns, IGA, referrals for medical care	– Partnered with St. Theresa's Hospital with support from traditional healers, local chiefs – Builds on African traditions of family support and mutual obligation	– Support from UNICEF – Support for nursing materials and drugs from the MOH	– HBC volunteers – Senior nurses, sisters & foreign doctors provide supervision	– Volunteers receive monetary compensation – Annual report for accountability	– Monthly review of HBC program – Monthly meetings of project members
Dignitas International Est. 2004 (Source: Dignitas International)	Africa: Malawi	To increase prevention, treatment and care, develop and disseminate solutions that harness community solutions – Stand up for those who lack access	– PLWHA, family members, community members, CHW, vulnerable populations (e.g. women, youth, soldiers, prisoners) – mostly rural with urban town centre	– Home, community – Distribute condoms, VCT, PMTCT, ART, adherence monitoring, treat and prevent OI and STIs, HBC, palliative care, psychosocial care, information, education and communication	– Collaborates with Zomba Central Hospital, District Health Office, Christian Health Association of Malawi, public health researchers, academic institutions & Malawian health authorities – District-level: population 670,000 – decentralization to 25 health centers – expansion to other districts	Donors include: – BMO financial group, Donner Canadian Foundation, MAC AIDS Fund, Peterborough KM Hunter Charitable Foundation, Sullivan Entertainment – Rotary International, Stephen Lewis Foundation – St Michael's Hospital Foundation – USAID	– Clinicians receive ongoing training through MOH – On-the-job training and support	– Knowledge translation through operations research team – Strategic plan – Expansion to other districts	– Regular monitoring – Publication in peer review journals – Annual reports
Family AIDS Caring Trust (FACT) and Family, Orphans and Children Under Stress (FOCUS) – FACT est. 1988 – FOCUS est. 1993 (Source: DeJong, 2001, FACT website)	Africa: Zimbabwe, Mozambique	To be a renowned, result-focused quality program that facilitates sustainable programs on mitigation of the impacts of HIV/AIDS To respond to the growing number of malnourished OVC	– PLWHA, OVC, family members & caregivers – urban and semi-rural settings	– Home, community – HBC, nutrition support, counseling, pastoral support, bereavement support, trains family in infection control, household assistance, material assistance (food, soap), legal aid & advice, workshops, referrals for medical care	– Incorporates church life – Works with church-based CBO, NGO, and ministries and institutions at the local, national, and international level – Multi district, parts of Mozambique – Focus on community mobilization	Donors include: – Save the Children (USA), UNICEF, Plan International (Mutare), Action AIDS International, CHF, Concern Worldwide, Healthlink Worldwide, Population Services International – Global Fund – European Commission	– Recruits CHW, enlists church members		– Evaluation to highlight gaps and areas for improvement – Evaluate impacts of workshops
Hope Worldwide Siyawela Community Child Care Est. 1999 (WHO, 2002)	– Africa: South Africa	To respond to growing numbers of OVC	– Children, OVC, immediate families and caregivers	– Home, community prevention education, VCT, PMTCT, HBC, nutrition, psychosocial support, victim support, recreational activities, life skills, food parcels, microfinance, community-based research project, referrals	– Partnerships with microfinance institutions, microenterprise development organizations, victim support groups, VCT& PMTCT specialists & preschools and Perinatal HIV Research Unit – bridge gaps between central hospital and 12 community health clinics – focus on community mobilization, community-based research and capacity building	– Volunteers	– Trains volunteers in child care and HBC	– overall program strategy	– Conducting participatory research to identify specific needs within the community – research findings feed into strategies to improve program operations
Khutsong Centre and Heartbeat orphan programme Est. 1998 (WHO, 2002) Save the Children 2006)	Africa: South Africa	To build trust	– PLWHA and their families – in a gold mining town	– Home – Poverty alleviation, IGA and support, training in life skills, food parcels	– Established by Carletonville AIDS Action Committee – partnerships continually developing (schools, churches, support groups) – partnership driven: within team and with ill people, families, community, and agencies and ministries – 4 communities	Donors include: – government ministries (Health, Social Development, Justice, Education and Agriculture) – mining company & bank – Save the Children UK	– Team: nurse coordinator, social worker, orphan care workers, nurses and caregivers – paid care-givers – local member of parliament sits on project committee	– Care-givers visit child headed households on a regular basis – developed comprehensive training program – handed over day-to-day running to Sakhi Sizwe Community Child Forum to focus efforts on establishing new programs	– Some M&E activities in annual Operational Report
Lusikisiki Clinic (Source: Bedelu et al., 2007)	Africa: South Africa	To deliver HIV care & services through decentralization, task shifting within services, training & mentoring staff, creating strong community support	– PLWHA – Rural	– Home, community – VCT, PTMCT, ART, HIV management, HBC, TB diagnosis, treat OI	– Sub district reach – Decentralization and task-shifting as service delivery model	– Support through MSF	– ARV program run through nurses & CHW, supported through regular physician support (including mobile teams) and adherence counselors – community caregivers mentored, nurse supervision – overseen by MSF	– Clinic committee represents users with complaints, advocate for better infrastructure, drug supply – quarterly evaluation of outcomes of HIV care – weekly meetings and workshops to build capacity	Monitoring and evaluation, quality control – clinical outcomes
Moretele Sunrise Hospice HBC Est. 1998 (WHO, 2002)	Africa: South Africa	– To address HIV/AIDS	– PLWHA, OVC, palliative patients, families – rural	– home – VCT, prevention education, HBC, nutrition support social support, disclosure support, bereavement counseling, food parcels, assistance with gardens, life skills, recreation programs, IGA, referrals	– 27 health clinics and 78 villages within 120km – inter-disciplinary approach	Donors include: – VSO – government – international	– Interdisciplinary team (palliative nurse, social worker, physician, pastor & psychologist) – supervisors trained to manage CHW – ongoing training and mentoring	– Community mobilization – satellite programs set up to provide services to those who live far	– Conduct research
Reach Out Mbuya Parish HIV/AIDS Initiative Est. 2001 (Source: Reach Out Mbuya website, Chang et al., 2009)	Africa: Uganda	– a community free of the spread of HIV and where persons with HIV/AIDS are living positively with an improved quality of life	– PLWHA, OVC, guardians, partners, families, poor and vulnerable – urban/rural	– home, community – VCT, HBC, ART, PMTCT, laboratory testing, pharmacy, OI, adherence support, TB tracing, psychological support, food support, community sensitization, IGA	– faith based community program focused on holistic model of care – multiple partners including several ministries and the Makerere University School of Public Health – 6 village catchment area, 1 main site, 3 satellites	Donors include: – Friend for Reach Out, Makerere School of Public Health, Kampala City Council – Barclay's Bank – PEPFAR, CDC, Medical Mission International, SidEcole, ROSE, Infectious Diseases Institute, Danish Group, Program for Accessible Health Communication and Education, Australian Embassy, Uganda Bikers Association – Global Fund, Stephen Lewis Foundation CARE Uganda	– Executive Director and 8 member Board of Directors – Chaired by Parish Priest – 258 staff (2009), 147 volunteers (local and international) – team leaders in each village works with team of community workers	– 5 year strategic plan – internal audits – on-going training for all staff	– department with Research Capacity and Capability Group – Annual Reports, presentations at conferences – patient tracking through electronic registry
Scaling Up Through Expanded -Partnerships (STEPS) Est. 1995 (Source: Kadiyala, 2004)	Africa: Malawi	– goal to mobilize sustainable community action to prevent the spread of HIV & mitigate the impact on OVC and families	– PLWHA, households, OVC, community members	– prevention, risk reduction, HBC – social support, poverty alleviation, food security for HIV-affected households, HIV advocacy, fundraise & resource mobilization, early childhood care support, life skills training	– Village AIDS committees identify the vulnerable, then plan responses – operates in 8 districts – multi-sectoral approach	Donors include: – Save the Children USA	– volunteers, teachers, community action groups mobilized	– develop subcommittees to build capacity (district, community and village level) – model adapted in Ethiopia, Mozambique, Zambia through partnerships and training by other NGO/CBO – strategic plan for financial sustainability	– manage, monitor & evaluate, accountable decision making process
Thandanani Children's Foundation Est. 1989 (Source: Thandanani website)	Africa: South Africa	To keep children in their own communities – name means ‘love one another’ in Zulu	– OVC, families – urban centre surrounded by peri-urban and semi-rural areas	– home, community – HBC, assistance with school fees, support & develop community-based Early Learning Centres, facilitate establishment of gardens, volunteer-driven IGA, establishment of saving groups, welfare assistance, referrals for medical care	– focus on advocacy & community mobilization – community leaders, residents, volunteers identify children who are abandoned, abused or at risk of being orphaned – reach: 16 communities	– Donors include: – South Africa Department of Welfare – Chevron (local) – Lawyers for Human Rights, Christian AIDS, Catholic Agency for Overseas Development, Stephen Lewis Foundation, Kindernothilfe, Belgium Embassy, CAFOD – financial report annual	– community residents, leaders, volunteers – Staff of 21, team of 109 volunteers – volunteers receive training and support – 5 person Board of Directors – Staff in 4 areas: Management and Administration; Development, Development and Direct child Support, Welfare and Early Childcare, Health	– hold regular volunteer meetings – staff and volunteers paid salary (highly dependent on economy)	– monitor regularly – produce Annual Report and articles on their programs
The AIDS Support Organization (TASO) (Source: TASO website) Est. 1987 largest indigenous NGO in Africa indigenous	Africa: Uganda	– A world without AIDS – founding based on principle that people were unified by common experiences faced with encountering HIV at a time of high stigma, discrimination and ignorance.	– PLWHA, families, communities	– home, community – prevention strategies, medical care, ART, HBC, outreach clinics, nutritional support, disclosure counseling, social support, material support, day care center, apprenticeship program, AIDS youth club, HIV advocacy	– partnership with MOH & other stakeholders, provides opportunities for trainees from other parts of Africa area – 11 service centers, 22 ‘mini-TASOs’ in regions outside catchment – build capacity	Donors include: – government Ministry of Health, Uganda AIDS Commission – Civil Society Fund, Danish International Development Agency, CDC, Swedish International Development Agency, UK Department for International Development, Irish AID	– membership organization governed by Board of Trustees – Regional Advisory Committees and Centre Advisory Committee to guide operations in different regions – staff, volunteers – training in community skills, specialized skills, information & knowledge in care. – training of the trainers	– strategic plan to reinvigorate prevention strategies through enhanced partnerships local, national, and international	– client views incorporated to provide feedback – operational research (formalizing internal research structure) – publication in peer-review journals
Tumelong Hospice and Lekegema Orphan Haven Est. 1999 (WHO, 2002)	Africa: South Africa	To let people die in peace – Tumelong is a Tswana word for ‘place of faith’ – provide orphans with holistic care	– PLWHA, most between 15–25, OVC and the communities where they live, grandparents, neighbors, and other orphan care givers – semi-rural (farmland) area	– home, community – HBC, palliative care, hospice, OI treatment, TB care, cancer care, bereavement counseling, material support, nursery, death certificate obtainment, counsels on inheritance rights & orphan grants, day care, weekend programs	part of larger Anglican mission (est. 1939) – holistic approach – reach: 1 million people in 8 townships	– Donors include: – government ministries – business (e.g. local supermarkets provide fresh and vegetables) – religious missions	– run by team of 18 lay CHW – team approach, staff, CHW, social workers, physician, nursing sister) – 1 worker/per 4–5 PLWHA – in-service training sessions	– regular meetings to provide mutual support – every 3 mo: staff has team-building break and families join in for recreational activities (transport provided) – plans for expansion	
Kapit Kamay Sa Bagong Pag-Asa (Source: WHO 2002)	Asia: Philippines	To mitigate impacts of HIV/AIDS	– PLWHA, families	– livelihood assistance, micro-finance	– partnership between Philippines National AIDS Council, Centre for Community Transformation and the Pinoy Plus Association Inc – fund proposals all over country		– train management	– rigorous standards established for reviewing and disbursing funds	– identifies problems with program (e.g. confusion over loan repayment, distance between projects hard to monitor) and seeks to address
Servants to Asia's Urban Poor (SERVANTS) Est. 1996 (Source: Disability Action Council website)	Asia: Cambodia	To improve the lives of the poor	– PLWHA, migrant, squatter communities, children with disabilities, OVC, women – urban slum, brothels	– home, community – prevention education, provide condoms, HBC, malnutrition clinic for children, immunization, women's health program, pain relief, brothel clinic, treat STI, TB, counseling & support, sanitation program, education	– district level reach partner with local AIDS committee, numerous collaborators/partners including Veterans International, Center for Child Mental Health, TB center, Pasteur	– Donors include: – ROTANAK Foundation, Tear Fund UK – individual donors	– volunteers	– plan to extend program – run refresher courses – stipend for volunteers, programs for staff & family to avoid burnout (recreational activities, health and social assistance)	
HIV Equity Initiative/ Zanmi –Lasanti (ZL) Est. 1998 (Source: Partners in Health, 2006)	Central America: Haiti	To provide care and treatment for HIV/AIDS	– PLWHA – in poorest country and worst HIV epidemic in western hemisphere	– community – prevention & risk reduction, VCT, PMTCT, ART, adherence monitoring, TB care, malnutrition, treat diarrhea, pneumonia, STI, reproductive care	– in 1998, ZL became world's first program to provide free, comprehensive HIV care and treatment – only NGO to provide comprehensive primary care – partners with other Haitian NGOs	– Donors include: – Global Fund to Fight AIDS, Tuberculosis and Malaria – strategic plan each year to review budget	– governed by Comite Executif – CHW or accompagnateurs supported by nurses, doctors, lab technicians, social workers	– compensation for volunteers, workers paid – project expanded to Peru, Russia, USA, Guatamala, Rwanda, Malawi, Lesotho, Burundi, Mexico, Kazakhstan, Dominican Republic	– extensive evaluation & dissemination of findings (reports, peer-review journals)
Project Hope-Projecto Esperanca Est. 1988 (legally constituted 1991) (Source: Family Health International, 2004)	South America: Brazil	Best therapy is to be with family, families should be helped to accept HIV, infected individuals and their families should be helped to demand & fight for their rights of citizens – motto: hand in hand in life	– PLWHA, caregivers of OVC, families – urban	– home, community – prevention & risk reduction strategies, HBC, nursing care, occupational therapy, emotional & psychological support, social programs, campaign for OVC, educational material & public talks aimed at specific groups including students, young people & housewives	– slum in city – partners with other 30 NGOs	– Donors: – local – Brazilian National AIDS Programme, – CAFOD-UK, Caritas-Holland, Austrian-DK	– sisters with support from local bishop – staff include paid staff, volunteers, godmothers/godfathers, casual workers – volunteers undergo orientation and training for 2 months	– incentives for unpaid staff include recreational activities, transport passes, month-long holidays – expanded to other centers	activity reports, monitoring – Produce manuals on community health worker curriculum and program management
Men as Partners Program Est. 1998? (Source: Engender Health website)	Global: 15 countries in Africa, Asia, Latin America and US	To mobilize men- to become actively involved in countering HIV and gender-based violence	– men as partners, women	– community – provide workshops on health and safety of men, women and child, incorporate gender, courage, activity and effects of HIV			– CHW and health care providers trained to provide workshops – ongoing training and technical assistance	– training manuals – to reach more men, build capacity within NGO sector, promote CB efforts to mobilize men – establish partnerships with Solidarity Centre, the AIDS Consortium, South African National Defense Force, Hope Worldwide, Perinatal HIV Research Unit at Chris Hani Baragwanath Hospital	– evaluation of pre- and post-workshops to determine change in attitudes
Pathfinder International Est. 1957 (Source: Pathfinder International website,	Global: 25 countries	– people everywhere should have the right and opportunity to live a healthy sexual and reproductive life	– PLWHA, orphans, youth – vulnerable groups: sex workers, injection drug users, displaced people, migrants, military workers	– home, community – VCT, counseling, ART, HBC, PTMCT, adherence support, OI prevention and treatment, TB prevention and care, family planning, training local volunteers, sensitization messages, referrals, IGA	– integrated HIV/AIDS care in early 1990's – reproductive health services as the center of all programs/services – partners with local, national, international partners	Donors include: – USAID, CDC, Swedish International Development Cooperation Agency, Brazil National AIDS Program, WB, United Nations Population Fund, Gates Foundation, McKnight Foundation, Trull Foundation, Ford Foundation, Tides Foundation	– Board of Directors, President – Senior leadership supports country representative – 752 field staff	– conduct studies to enhance program learning – multi-disciplinary Research and Metrics Staff build capacity for data-based evaluation for program learning Building – strengthen capacity of local organizations	– evaluation strategy, measure key indicators – performance monitoring produce annual reports – peer review publications

Gaps reflect incomplete data; Definitions: AIDS, Acquired Immune Deficiency Syndrome; ARV, antiretroviral; CAFOD, Catholic Overseas Development Agency; CB, community-based; CBO, community-based organization; CDC, Centers for Disease Control; CHW, community health worker; HBC, home-based care; HIV, human immunodeficiency virus; HTC, HIV testing and counselling; IGA, income generating activities; MSF, Medecins Sans Frontieres; MOH, Ministry of Health; NGO, non-governmental organization; OI, opportunistic infection; OVC, orphans and vulnerable children; PEPFAR, Presidents Emergency Plan for AIDS Relief; PLWHA, people living with HIV/AIDS; PMTCT, prevention of mother-to-child transmission; STI, sexually transmitted infection; TB, tuberculosis; VSO, Voluntary Services Overseas; UNICEF, United Nations Children Fund; USAID, United States Agency for International Development; WB, World Bank; WHO, World Health Organization.

### Region

CBC programs can be classified according to the geographic region in which they operate. While some programs, for example, ‘Men as Partners’ and ‘Pathfinder International’, operate globally, most programs we identified only work in one country, for example, Moretele Sunrise Hospice operates only in South Africa.

### Vision

Most of the included CBC programs described their program vision. This can be very broad such as the vision of The Aids Support Organization (TASO) of ‘a world without AIDS’ or Dignitas’ pledge ‘to stand up for those who lack access’. Most included programs provided a mission statement to describe what they seek to accomplish. Examples include the Centre for Positive Care ‘to reduce the spread of Sexually Transmitted Infections (STIs), HIV/AIDS, and to improve the quality of life’; SERVANTS to ‘improve the lives of the poor’; or Men as Partners to ‘mobilize men to become actively involved in countering HIV and gender-based violence’.

### Characteristics of target population

Urban–rural location can influence program delivery options and resources. The majority of CBC programs in this study described working with urban populations (e.g. Catholic Diocese in Ndola, SERVANTS) although semi-rural (e.g. Tumelong Hospice) and rural (e.g. Chirumhanzu HBC) settings were also represented. A few included programs serve both rural and more urban populations (e.g. FACT). While CBC programs focused on HIV seek to work with PLWHA, many programs also cater to others affected by HIV – family members (e.g. Centre for Positive Care) and orphans and vulnerable children (OVCs) (e.g. FACT/FOCUS, Hope Worldwide Siyawela Community Child Care). Less common were programs that work with high-risk populations such as prisoners or migrants (e.g. Dignitas International, SERVANTS). Pathfinder International specifically described targeting marginalized populations, including drug users, sex workers, and displaced persons.

### Program scope (services provided)

Two broad categories were judged relevant here – location and types of services provided. In terms of location, some programs only describe operating directly inside the private homes of their clients through home-based care (e.g. Moretele Sunrise Hospice HBC) while others report operating in more shared community settings (e.g. Men as Partners, Reach Out Mbuya). Most commonly, programs provide care both inside and outside of the home, with some aiming for multiple locations, including a clinic or hospital (e.g. Lusikisiki Clinic).

CBC programs differed most on the range of services provided as we noted heterogeneity regarding types of services provided over different time points along the continuum of care. Common services reported include HIV prevention, including risk reduction activities (e.g. Centre for Positive Care, STEPS); HIV Testing and Counseling (HTC) (e.g. HIV Equity Initiative); Prevention of Mother-to-Child Transmission (PMTCT) (e.g. Centre for Positive Care); ART provision and follow-up, including adherence monitoring (e.g. Dignitas International, Lusikisiki Clinic); medical and nursing support, including treatment of STIs and management of symptoms and opportunistic infections (e.g. Catholic Diocese in Ndola, Centre for Positive Care, Reach Out Mbuya); nutrition support and food supplementation (e.g. FACT); palliative care (e.g. Tumelong Hospice and Lekegema Orphan Haven); financial support in the form of income-generating activities (IGA) or community funds (e.g. Khutsong Centre and Heartbeat Orphan Programme); material support, including technical support; information, communication, and education activities (e.g. AIDS Foundation, Chirumhangu HBC project, Pathfinder International); and psychosocial support (e.g. Catholic Diocese in Ndola). Such heterogeneity is at the core of challenges involved in evaluating outcomes of such programs.

### Program operations

A number of important characteristics relate to the manner in which programs situate themselves in relation to community structures. First, we found that programs can be classified by the extent of community engagement and/or participation. In some CBC programs, community leaders or volunteers are included in overall operations management (e.g. Catholic Diocese in Ndola). Almost all programs where detail was provided implied the need to maintain and incorporate community life. For example, by seeking to build on traditions of family support, Chirumhanzu HBC project encourages a mutual obligation in the way they run their programs and deliver their services. Programs like FACT and Reach Out Mbuya incorporate church life, and in programs like the Thandanani Children's Foundation, it is the community leaders and the residents that primarily identify at risk individuals in need of their services. The number of partners and collaborators not only affects program operations but also may reflect a program's ability to build capacity in the broader community. In terms of program reach, not all included CBC program descriptions had available data, but among those that did, most have at least sub-district (e.g. Luskikiki clinic) or district-level reach (e.g. Dignitas International). Programs like TASO, STEPS, and Moretele Sunrise Hospice HBC serve patients in more than one district and can be considered multisite. To achieve coverage, programs decentralize (e.g. Dignitas International, Lusikisiki Clinic) or, through advocacy, they mobilize communities (e.g. Thandanani Children's Foundation). Finally, the extent to which CBC programs are embedded within formal health and social structures varies. Most programs reported linkages and collaborations with local organizations and government structures/ministries (e.g. Tumelong Hospice, Pathfinder International, Lekegema Orphan Haven). However, some of the programs described operate independently (e.g. HIV Equity Initiative/Zamni-Lasanti).

### Funding models

Financial aspects of the program are crucial – identifying who funds the program and how the funds are disbursed. Sources of funds described ranged widely between programs, with common sources including a combination of local and foreign governments (e.g. Chirumhanzu HBC, Khutsong Centre, and Heartbeat Orphan Programme), private donors (e.g. AIDS Foundation, Dignitas International), national or international NGOs (e.g. Centre for Positive Care, STEPS, Dignitas International), and individuals and businesses within the community (e.g. Khutsong Centre and Heartbeat Orphan Programme, Tumelong Hospice, and Lekegema Orphan Haven). Program costs and budgets were generally not provided in the sources examined. A few programs, such as Pathfinder International, did make their annual financial reports that documented expenses publicly available.

### Human resources

The organizational structure and staff composition of the CBC programs varied considerably, particularly with respect to the type of CHWs involved (e.g. nurses, clinical officers). The exact hierarchical structure was described only in a few programs (e.g. Pathfinder International), although most provided information on the presence of a board of directors (e.g. AIDS Foundation, TASO), advisory boards (e.g. Khutsong Centre and Heartbeat Orphan Programme, TASO), or other governance groups (e.g. HIV Equity Initiative). Some programs only described recruiting volunteers (e.g. SERVANTS) although more common were programs where a combination of paid staff and volunteers were recruited (e.g. HIV Equity Initiative, Lusikisiki Clinic). The methods, frequency, and level of training also ranged across included programs, as did the extent of volunteer and staff supervision or mentoring. When described, common strategies included on-the-job or ongoing training (e.g. Moretele Sunrise Hospice) or the provision of training sessions on specific topics (e.g. Project Hope, TASO). Among programs where information was available, management and supervision levels also varied although included programs commonly described incorporating a coordinator to supervise volunteers (e.g. Catholic Diocese in Ndola, Centre for Positive Care). Foreign doctors or senior nurses also provide supervision in programs such as Dignitas International, Lusikisiki Clinic, and HIV Equity Initiative. Some programs describe peer-based activities, including Pathfinder International and TASO's ‘training of the trainers’ program.

### Sustainability

According to our taxonomy, key factors influencing the sustainability of CBC programs include the availability of ongoing funding, the ability of the program to adapt to local contexts and to replicate itself in new settings, and high staff retention levels. According to the included programs, strategies used to retain staff include providing volunteers with a stipend or other incentives such as recreational activities and family support (e.g. Centre for Positive Care, SERVANTS, Thandanani Children's Foundation). Also, important for sustainability of programs is their ability to adapt to any changing local contexts and situations and, where appropriate, their replicability in new settings. We found few programs describing such replication; examples include STEPS and the HIV Equity Initiative. Dissemination, capacity building, and knowledge translation activities were described in several programs (e.g. Dignitas International, TASO).

### Monitoring and evaluation

Finally, the frequency and comprehensiveness of monitoring and evaluation activities varied. Programs such as the Catholic Diocese in Ndola described conducting biannual evaluations although specific details regarding these evaluations were generally not provided. Some programs provided quality indicators (e.g. AIDS Foundation, Lusikisiki Clinic, Kapit Kamay Sa Bagong Pag-Asa) or potential program impacts (e.g. TASO, Men as Partners) and have a specific team for evaluation and monitoring activities (e.g. Pathfinder International). Inputs, such as management structures and financial accountability (e.g. STEPS), and outcomes, such as changes in behaviors (e.g. TASO, Men as Partners) and clinical outcomes (e.g. Lusikisiki Clinic), were also described with respect to monitoring and evaluation activities.

## Discussion

The taxonomy presented here elaborates nine key categories useful for describing and organizing CBC programs focused on HIV/AIDS in resource-limited settings. Our findings can help to identify what is currently being done with respect to addressing the needs of PLWHA, highlighting any potential gaps in programming.

### Key characteristics of identified CBC programs

The majority of CBC programs included in our review described programs operating in sub-Saharan Africa, which is consistent with previous literature (9, 48).

Visions and program missions varied, although generally identified CBC programs strive to improve the lives of those living with HIV/AIDS. The specificity of the program's mission not only impacts upon the types of services offered but can also affect a program's ability to expand and adapt into new settings. While the vast majority of included programs targeted PLWHA, many also incorporated other affected populations, including family members and OVCs. A few CBC programs included vulnerable populations, such as drug users and migrant workers. Marginalized populations remain a priority population for HIV care, treatment, and support although they are generally underserved (16). Few programs provided data on the gender make-up of the populations being served. Further detail on the number of men and women receiving services would provide meaningful data in terms of coverage and equity of access to services. Currently, there is an absence of gender-specific data on patient-retention in ART programs and as a result there is conflicting evidence as to whether men or women are more likely to access ART (54, 55).

The types of services offered varied considerably among the CBC programs studied, although most described providing services both in the home and in more public community settings. Interestingly, in a review of South African CBC models, four common program types emerged: 1) funding, technical assistance, and support; 2) counseling, education, and an IGA component; 3) the two aforementioned points plus home visits; and finally, 4) comprehensive programs which add to item 3) above, additional levels of nursing care (17). Adding the location of services provides additional insight as to where the majority of programs provide care as well as where there may be gaps. Not surprisingly, almost all included programs provided care both in the home and in the community. Relatively few provided care only in the community or only in the home. Further assessment may help to identify services that are not offered. Marginalized groups, including injection drug users, may require other service locations or additional services, including harm reduction approaches.

Operational models also differed across programs included in the present review. In particular, the level of community engagement appeared to vary, as evidenced by the number and nature of collaborations and partnerships with community groups, members, and leaders. Importantly, the ability to build and maintain collaborations with community partners is essential to ongoing program operations. Related is how CBC programs are embedded within existing formal structures which include: the identification of community partners for potential collaboration, a sense of ownership by the community, their relevance to local needs, integration into existing systems, and periodic reviews and program updates with new knowledge (56). The availability and comprehensiveness of existing health and/or social structures and institutions (e.g. hospitals, schools, businesses, and religious structures) may also affect the ability of CBC programs to engage in collaborations and may be a function of multiple other factors, including available funding. Further identification of how contextual factors such as local politics, political interests, economic reforms, and various forms of conflict can impact on the capacity of CBC programs to perform effectively and sustainably may be needed.

Funding models, human resources factors, and the sustainability of CBC programs have also been included as part of our taxonomy and are all essential elements of program impacts. Interestingly, a recent review noted that factors influencing HBC program effectiveness include those that relate to human resources, funding mechanisms, and an ability to adapt to local contexts (37). Furthermore, sustainable project implementation has been identified as key for the scale-up and expansion of comprehensive HIV services in resource-limited countries (37, 57, 58). While significant elements of sustainability [identified by Torpey et al., 2010 (57)] include technical, programmatic, social, and financial forms of sustainability, in the present study, we found that the sustainability of programs is primarily related to the availability of ongoing funding and an ability to retain staff (57). It may be worth noting that models may be challenged to involve community members fully in decision-making regarding program goals and activity planning specifically when the majority of funding comes from a single source based outside the community (23). Current challenges related to funding, including the suspension of the Global Fund to Fight AIDS, Tuberculosis, and Malaria Round 11, holds very real implications for program sustainability. According to a Médecins Sans Frontieres (MSF) briefing note, several African countries have been forced to scale back on their programming (e.g. initiating new patients on ART) given budget and financial constraints (59). With respect to staff retention, there remains a need to identify sustainable compensation methods that are equitable and fair. An ability to ensure minimal necessary staffing levels as well as ongoing training and mentorship are also key program challenges (28) and yet are critical for program effectiveness and overall sustainability. Knowledge translation also plays an important role as the dissemination of successful strategies may be particularly useful for the long-term sustainability of programs as well as their scale-up and adoption other settings (60).

While most programs make reference to some type of regular monitoring, many programs provided limited information regarding their monitoring and evaluation strategies or the information that was collected during their evaluations. However, CBOs are playing greater roles in both the design and implementation of research in order to effectively inform program and policy decisions (16, 61). This underscores the need for adequate documentation and dissemination. Programs like TASO in Uganda capture regular data on clinical (e.g. proportion of patients with optimal adherence) and behavioral outcomes (e.g. disclosure of status, changes in community support) (62).

### Limitations

Several limitations must be considered when interpreting this review. First, the comprehensiveness of our review is limited because many CBC programs do not publish or make vital information publicly available. As our search strategy relied heavily on electronic resources, our review contains only those programs with access and capacity to publish their program details online. Even when data were available, not all CBC programs were included as we sought to represent typical, known programs. This may affect the generalizability of our taxonomy. More specifically, programs that solely target marginalized groups, including men who have sex with men, sex trade workers, and/or migrant populations, were underrepresented in our review. However, we did attempt to include a range of programs which, in addition to targeting the general population of PLWHA, targeted marginalized groups as well (e.g. Pathfinder International, AIDS Foundation). Furthermore, CBC programs originated from several regions, though not all. Nevertheless, the present study helps to disentangle general versus country-specific characteristics of CBC programs (38, 63). Finally, as we did not contact the programs directly to obtain additional information, we may have missed important details or descriptions that would add to the comprehensiveness of our taxonomy. Case studies of individual CBC programs often differed in the information provided, and the limited detail regarding the methodologies used (e.g. sampling, biases) in non-peer-reviewed data included in this review make it difficult to assess the validity of these findings. Partly for this reason, we did not assess the quality of included reports.

### Implications of presented taxonomy

**Fig. 1:**
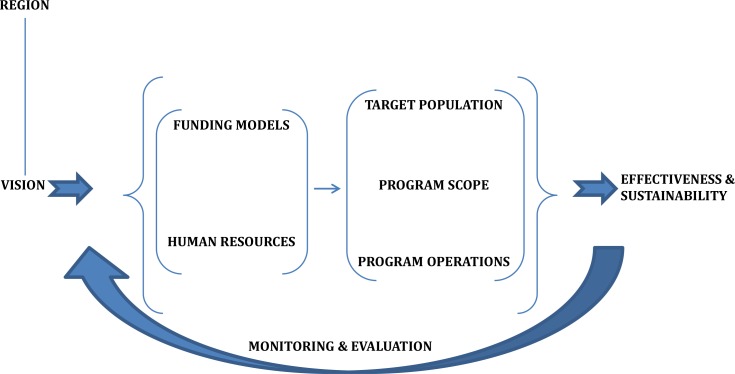
Potential program logic model for effective and sustainable CBC programming as informed by our presented taxonomy.

Huge sums of money are now being invested into multi-sectoral approaches and there have been extensive efforts to assess the effectiveness of traditional public health interventions in terms of prevalence rates and numbers of infections averted (64). Currently, mitigation indicators are not capturing data on the impacts of programs themselves but rather tend to focus generally on inputs and outputs (i.e. number of orphans schooled, number of individuals tested) (64). In Fig. 1, we present a potential logic model based on our taxonomy for CBC programs that may guide analyses of factors relevant for program effectiveness and sustainability.

In brief, through a clear vision, a need for a program is identified. This overall vision or mission helps to motivate and guide program development, including the specification of a target population and program scope. The inputs to the program include available funding and human resources. These inputs are needed to provide the target population a certain set services (program scope) using a specific approach (program operations). These can be considered our outputs. The number of households captured or the number of eligible patients initiated on ART offer some examples of outputs that could be described. The overall program outcomes would be effectiveness, the degree to which the program is meeting its goals (65), and sustainability. However, adequate monitoring and evaluation activities which feedback into all components of our model is fundamental for overall program performance.

Arguably, continuous feedback and quality improvement through regular monitoring and evaluation can facilitate any recommended rapid changes in the program to improve functioning and effectiveness (13). Evaluation guidelines with clear indicators of success informed by collaborations with various partners and drawn from governmental or ministerial standards, guidelines, and operating procedures (57) are required to ensure that programs are achieving their goals and objectives. Future research that seeks to develop a taxonomy of evaluation indicators useful for assessing CBC program effectiveness may be needed. Evaluation tools should include various process and output indicators, both broad and specific, and should be able to collect data on both patient-level outcomes as well as program outcomes (65).

Important considerations include whether programs have identified resources for monitoring and evaluation activities. Improved methods for data collection, clearly defined indicators of success, and well-maintained ongoing monitoring and evaluation systems are crucial for program planning and effective implementation (48, 66, 67). However, gaps in sustainable evaluation research capabilities have important implications with respect to the level of research that can be undertaken in resource-limited settings (68). An operations research approach which considers real world conditions, including limitations, in the number and time of trained health workers and/or challenging research environments may offer a particularly useful method for conducting key monitoring and evaluation activities in such settings (69).

## Conclusions

High costs and a lack of adequate health infrastructure can challenge the scale-up and uptake of HIV-related services in resource-limited settings. Chronic health worker shortages have resulted in the decentralization of HIV care and a movement toward the use of non-physician-based models of care (14, 28, 70). CBC programs can encourage partnerships among different stakeholders and sectors and build on the supportive community networks that already exist for PLWHA (9).

Our taxonomy, focused on HIV/AIDS in resource-limited settings, classifies CBC programs by nine key areas: region, vision, target populations served, program scope, program operations, funding models, human resources, sustainability, and monitoring and evaluation strategies. While further study is needed, our findings can provide insight on current CBC models as well as potential gaps in programming. Furthermore, the presented taxonomy can inform potential logic models that can be used to enhance overall program performance. In the context of ART scale up, our findings have potential for use in the development of evidence-based tools for sustainable HIV/AIDS-service delivery in regions currently facing, or at risk for, severe health resource shortages.
